# Adverse Environmental Impact: 30-Year Search for a Definition

**DOI:** 10.1100/tsw.2002.166

**Published:** 2002-03-08

**Authors:** David A. Mayhew, Paul H. Muessig, Loren D. Jensen

**Affiliations:** EA Engineering Science and Technology, Inc, Hunt Valley, MD 21031, USA

**Keywords:** adverse environmental impact, Clean Water Act, Section 316(b), best technology available, cooling-water intake structure, entrainment, impingement, public trust resources

## Abstract

Since passage of the Clean Water Act in 1972, there has been a long, unresolved struggle to define a key phrase in Section 316(b) of the act: “adverse environmental impact” (AEI). Section 316(b) requires that the best technology available be used in cooling-water intake structures to minimize AEI due to entrainment and impingement of aquatic organisms. Various attempts were made to evaluate and define AEI, including focused national conferences on impact assessment. Unresolved arguments regarding AEI were reinvigorated following the 1995 Consent Decree requiring EPA to propose new rules to implement Section 316(b). This article reviews and compares eight proposed definitions of AEI. Six of the definitions define AEI as impact expressed at the population or higher level of biological organization. The two remaining definitions are unrelated to populations: a 1% cropping of the near-field organisms and “one fish equals AEI”. The latter definition is based on the desire of some stakeholders to define AEI as the loss of any public trust resources. Equating loss of public trust resources with AEI hampers consensus on a definition because a societal-based policy concept (public trust resources) is commingled with science-based definitions based on population effects. We recommend that a population-based definition of AEI be incorporated into Section 316(b) guidance and observe that this will not preclude a state from exercising its law and policy to protect public trust resources.

## INTRODUCTION

Soon after passage of the National Environmental Policy Act in 1969, which brought the term environmental "impact" into common usage, the U.S. Congress passed Public Law (PL) 92-500, the Federal Water Pollution Control Act Amendments of 1972 (the "Clean Water Act" or "CWA"). Section 316(a) of the CWA addressed thermal discharges, and Section 316(b) addressed cooling-water intake structures (CWIS). Section 316(b) required that "the location, design, construction, and capacity of cooling water intake structures reflect the best technology available [BTA] for minimizing adverse environmental impact." Such impact can result from entrainment of fish eggs and larvae and other small aquatic organisms into the cooling-water stream (and ultimately through pumps and condensers) or from impingement (trapping) of larger organisms on CWIS screens. Although possibly not the first use of the phrase "adverse environmental impact" (AEI), its incorporation in the federal law solidified it as a litmus test in subsequent CWIS impact assessments. Unfortunately, the phrase was not defined or quantified, and this resulted in much confusion, controversy, and litigation. The confusion has continued. Now, 30 years after passage of the act, and after considering four possible definitions in its draft rulemaking for new CWIS (Federal Register Vol. 65, No. 155, pp. 49060-49121, 10 August 2000), the U.S. Environmental Protection Agency (EPA) declined to define AEI in its final rulemaking of 9 November 2001 (Federal Register Vol. 66, No. 243, pp. 65256-65345, 18 December 2001).

## HISTORY

It did not take long after passage of the CWA for scientists, regulators, and resource managers to begin to grapple with the meaning of AEI. In June 1975, a *Conference on the Biological Significance of Environmental Impacts* was sponsored by the U.S. Nuclear Regulatory Commission and held at the University of Michigan[1]. The consensus definition that emerged from this forum was:

> An impact is significant if it results in a change that is measurable in a statistically sound sampling program and if it persists, or is expected to persist, more than several years at the population, community, or ecosystem level[2].

The word "adverse" was not featured in this forum, but we equate it with the word "significance."

Soon after the conference, the EPA published the 1977 *Draft Guidance for Evaluating the Adverse Impact of Cooling Water Intake Structures on the Aquatic Environment*[3]. This guidance contained the following definition of AEI:

> Adverse aquatic environmental impacts occur whenever there will be entrainment or impingement damage as a result of the operation of a specific cooling water intake structure. The critical question is the magnitude of any adverse impact. The exact point at which adverse aquatic impact occurs at any given plant site or water body segment is highly speculative and can only be estimated on a case-by-case basis by considering the species involved, magnitude of the losses, years of intake operation remaining, ability to reduce losses, etc.

Whereas the first sentence of this definition appears to identify any entrainment or impingement as adverse impact, it becomes clear that entrainment and impingement losses are not, in and of themselves, adverse impact, pending evaluation of various other factors.
In its 1980 strategy document for addressing power-plant impacts[4], the U.S. Fish and Wildlife Service defined impact as:

> A change in population structure or dynamics of a species resulting from an activity of man that remains at least as long as the activity continues.

Also in 1980, Voigtlander[5] reviewed prior attempts at defining AEI and proposed the following definition:

> An impact is a significant, long-lasting, man-induced change in the numbers or biomass of a species population.

Voigtlander also highlighted a fundamental problem with the concept of impact that has hampered consensus: "Obviously it [impact] is one of those words that is so familiar to us that we all understand what it means — except that everyone understands it somewhat differently." Similar observations were made by Westman[6] and several participants in EPA-sponsored public meetings on the 316(b) rulemaking (comments available at http://www.epa.gov/ost/316b/).

The four definitions above differ somewhat, but in each, the test of impact (or adverse impact or significant impact) pivots on a level of organization above the individual fish or other organism. Either explicitly[2,4,5] or implicitly[3], that level of organization is at least the population level. That is, impact is not deemed adverse or significant unless it is expressed and measurable at least at the population level.

The longest and most intense effort to identify impacts of CWIS took place on the Hudson River between the mid-1960s and 1980[7,8]. Fishing and conservation interests were concerned that entrainment of striped bass eggs and larvae at several power plants and the proposed Cornwall pumped-storage facility would harm the population. There was also concern regarding the loss of fish due to impingement at CWIS. Detailed field studies, population modeling, and other evaluations were conducted and then debated in a series of adjudicatory hearings. Ultimately, settlement negotiations were held wherein disputes over environmental impacts were suspended and replaced with a series of consensus mitigation programs. The mitigation agreements include ongoing monitoring and preparation of annual year-class reports and special entrainment and impingement studies. In the context of this article, the Hudson River studies were never directed at defining AEI as a regulatory standard or threshold. Rather, the effort was directed at measuring the effectiveness of mitigation measures in reducing mortality rates.

Similar long-term impact assessments were carried out at the Salem Nuclear Station on Delaware Bay between the early 1970s and mid-1990s. These studies and regulatory reviews culminated in the mid-1990s with a negotiated settlement with state and federal regulators based on habitat enhancement to offset CWIS losses and testing of alternative intake technologies to reduce impingement. Although AEI was not defined, the settlement was based on providing opportunities for increased biological production within the estuary to offset losses associated with operation of the CWIS at Salem. The cornerstone of the settlement was the utility's establishment and funding of the Estuarine Enhancement Program (EEP). The terms of the settlement were incorporated into the New Jersey Pollutant Discharge Elimination System permit issued in 1995. The primary component of the EEP consisted of restoration, enhancement, and/or preservation of more than 20,000 acres of degraded coastal wetlands and upland buffer along the Delaware Estuary; these wetlands provide nursery, food, shelter, and habitat for many species of fish affected by the CWIS as well as other wildlife. The EEP also included construction of fish ladders to enhance river herring migration and production, installation of protective intake technologies, and a comprehensive biological monitoring program. The EEP was retained in Salem's permit for 2000.

After the mitigation-based negotiated settlements on the Hudson River and at Salem, discussion of the meaning of AEI was reinvigorated following the 1995 Consent Decree with Hudson Riverkeeper et al., requiring the EPA to propose rules to implement Section 316(b). As evidenced in EPA-sponsored public meetings on the pending rulemaking and ultimately in the proposed rule for new facilities, several definitions of AEI were considered for possible inclusion in regulations or guidance.

## THE PRESENT

Two of the definitions in EPA's proposed rulemaking focused on population- or higher-level impacts. One was the same definition previously published in 316(b) guidance[3] and cited above. The second definition would place AEI in a biocriteria context, whereby CWIS affects on an aquatic community would be compared to a reference site without a CWIS. Presumably, measures (metrics) of community abundance, diversity, and other characteristics would be compared between the sites, and if similar, a lack of AEI to the aquatic community at the CWIS site may be concluded. An implementation approach was not provided by the EPA, but comments were invited.

Two additional definitions in the proposed rulemaking diverged from all previous definitions in that they were not related to population-level effects. One of these defined AEI as:

> impingement or entrainment of one (1) percent or more of the aquatic organisms from the area around the cooling water intake structure from which organisms are drawn onto screens or other barriers at the entrance to a [CWIS].

EPA considered this a "reasonable approach" because it was similar to its approach with water quality-based regulatory programs. We consider this a poor approach in that an AEI threshold is arbitrarily *assigned,* and no correlation to environmental damage or AEI was presented.

Another alternative considered by the EPA was to define AEI as "any impingement or entrainment of aquatic organisms." This has been informally referred to as the "one fish equals AEI" definition. In discussing this alternative in the proposed rulemaking for existing facilities, EPA cited public comments by a New York State Department of Environmental Conservation representative regarding its long-term implementation of this definition. In those public comments (available at http://www.epa.gov/ost/316b/), the New York State representative explained that agency's rationale for the approach, including, in part, the statement that "these are our trust resources as states, and we do not feel that [it] is right to allocate any of these resources to industrial mortality." Without debating the concept of trust resources, which has basis in law[9], this definition is unrelated to environmental damage or AEI. Furthermore, under such a definition, no CWIS could be permitted without maximum application of BTA, since none can totally avoid some level of entrainment and impingement—regardless of BTA employed.

Under the trust resources concept, the impingement of one fish during a year would represent AEI. At least outside of the context of threatened and endangered species, no one would construe the loss of a single fish as environmental or ecological damage. The idea of a state's ownership of natural resources—and the intrusion of this concept into the 316(b) process—is not new. For example, during a panel discussion at the *Fourth National Workshop on Entrainment and Impingement* in Chicago in 1977[10], a representative of the state of Michigan made a strong case for the state's ownership of the resources and stated, "even though the losses of fish do not warrant the application of extremely expensive technologies, we feel that we cannot let the utilities off for killing fish that belong to the state." In this discussion, the Michigan representative separated implementation of the federal 316(b) statute from a state's right to "mitigate" for losses of its resources. However, we believe there is a tendency in some areas to substitute any loss of a state's trust resources as a definition for AEI.

The Public Trust Doctrine is a legal concept that has its roots in the Roman Empire and which has evolved into a mechanism to protect natural resources for the public good[11]. The doctrine is considered a legal framework for resource planning and management that has increasingly been used not only to protect natural resources for public use but also to prevent overexploitation of those resources[12]. We do not dispute the public trust concept in general or its potential application in matters of CWIS impacts. However, we do not believe it is appropriate to substitute the protective concept of the doctrine as a definition of AEI. Some people may construe the loss of one fish as a social impact, i.e., a loss of public property. But it is not an environmental impact, and that is the focus of Section 316(b).

In response to the proposed rulemaking for existing facilities, the Utility Water Act Group[13] provided extensive comments, including a proposed definition of AEI:

> Adverse environmental impact is a reduction in one or more representative indicator species that (1) creates an unacceptable risk to the population's ability to sustain itself, to support reasonably anticipated commercial or recreational harvests, or to perform its normal ecological function and (2) is attributable to the operation of the cooling water intake structure.

This is another population-based definition, but it is unique in that its "test" or determination of threshold turns on not just a reduction in population, but whether that reduction represents "unacceptable risk." Further, it appears to address resource allocation issues in that unacceptable risks to fishery harvests may represent AEI outside of the context of population sustainability. The Utility Water Act Group proposed that unacceptable risk be determined in a scientific risk assessment and risk management process wherein a number of biological and social factors would be considered.

On 9 November 2001, a final 316(b) rulemaking for new CWIS was signed. After 30 years of research and debate on the meaning of AEI, the EPA declined to define it, citing the same lack of consensus among stakeholders as described in this article. The EPA assumed that entrainment and impingement were real or potential threats to aquatic populations and formulated the rulemaking as a technology-based approach for minimizing any entrainment or impingement.

## DISCUSSION

From the period of the early 1970s to the present, eight definitions of AEI were found in the available record and reviewed (Table 1). Six of these cast AEI in a population- or higher-level context. That is, the impact must be measurable and expressed at the population or higher (e.g., community) level of biological organization. Two of the newer definitions originally considered by EPA—"one fish equals AEI" and a 1% cropping of the nearfield waterbody population — were based on counts of entrained and impinged organisms. Whereas no one should argue the right of any stakeholders to consider these last two definitions, their inclusion in the suite did not make the achievement of consensus any easier. Prior to the 1990s, efforts to define AEI had a common basis—impact at the population (or higher) level of biological organization. Now, there is no common basis among competing definitions of AEI. The various definitions reviewed herein reflect the different values (scientific vs. social) of the various stakeholders involved.

In our view, the failure to define AEI in the final rulemaking for new CWIS will not end the debate. As the rulemaking process moves to consideration of the existing CWIS facilities, there will be renewed calls for inclusion of AEI in the process. Many existing facilities have substantial environmental data sets that can be used to determine the presence or absence of AEI. The EPA's rationale for not defining AEI — essentially that it is indefinable — is not compelling. We acknowledge that even among scientists, differences exist regarding what level of loss of aquatic resources represents damage or impact. This need not preclude establishing a definition based on population-level impacts, in the sure knowledge that the state of science will improve to be able to measure those impacts. Our position is that whereas AEI may not presently be easily measured, it is certainly definable.

In our review of historical and current discussions about AEI, we identified several factors that we believe are important, some of which have seriously hampered consensus on AEI.

**TABLE 1**
**Chronology of 316(b) and AEI Definition Milestones**

| Date | MileStone | Definitions |
|---|---|---|
| 1969 | Passage of National Environmental Policy Act; term "impact" comes into common use | |
| 1972 | CWA Section 316(b); term "adverse environmental impact" codified | |
| 1975 | Conference on *Biological Significance of Environmental Impacts*[1] | An impact is significant [adverse] if it results in a change that is measurable in a statistically sound sampling program and if it persists, or is expected to persist, more than several years at the population, community, or ecosystem level[2]. |
| 1977 | EPA (1977) Draft 316(b) guidance[3] | Adverse aquatic environmental impacts occur whenever there will be entrainment or impingement damage as a result of the operation of a specific cooling-water intake structure. The critical question is the magnitude of any adverse impact. The exact point at which adverse aquatic impact occurs at any given plant site or water body segment is highly speculative and can only be estimated on a case-by-case basis by considering the species involved, magnitude of the losses, years of intake operation remaining, ability to reduce losses, etc. |
| 1980 | Hudson River case settlement; culmination of the most studied and contested 316(b) issue | |
| 1980 | U.S. Fish and Wildlife Service power-plant impact strategy document | A change in population structure or dynamics of a species resulting from an activity of man that remains at least as long as the activity continues[4]. |
| 1980 | Fifth National Workshop on Entrainment and Impingement: *Issues Associated with Impact Assessment*[14] | An impact is a significant, long-lasting, man-induced change in the numbers or biomass of a species population[5]. |
| 1988 | Publication of AFS Monograph 4: Science, Law, and Hudson River Power Plants, a Case Study in Environmental Impact Assessment[8] | |
| 1995 | Consent Decree between Hudson Riverkeeper et al. and EPA requiring new Section 316(b) rulemaking | |
| 2000 | EPA proposed rule for new CWIS facilities (Federal Register, Vol. 65, No. 155, pp. 49060-49121, 10 Aug. 2000) | <u>Considered by EPA:</u><br>1) The definition from the 1977 316(b) guidance (see above);<br>2) Biocriteria-based definition (see text);<br>3) Impingement or entrainment of one (1) percent or more of the aquatic organisms from the area around the [CWIS] from which organisms are drawn onto screens or other barriers at the entrance to a [CWIS];<br>4) Any impingement or entrainment of aquatic organisms.<br><u>Utility Water Act Group[12] definition in response to proposed rule:</u><br>Adverse environmental impact is a reduction in one or more representative indicator species that (1) creates an unacceptable risk to the population's ability to sustain itself, to support reasonably anticipated commercial or recreational harvests, or to perform its normal ecological function and (2) is attributable to the operation of the cooling water intake structure. |
| 2001 | Final rulemaking for new CWIS, 9 November 2001 (Federal Register Vol. 66, No. 243, pp. 65256-65345, 18 December 2001). | No definition. Default assumption that any entrainment or impingement is threat to aquatic resources. |

1. Given the use of the phrase “adverse environmental impact” in Section 316(b) of the CWA and the extant disagreement over the meaning of the phrase, there should be a definition in regulation and/or guidance. Failure to do so would invite continued confusion and could lead to extended litigation among stakeholders regarding Section 316(b). Notwithstanding the lack of a definition in the final rulemaking for new facilities, there will be ample opportunity to resolve and define AEI as the 316(b)-rulemaking process continues.
2. Whereas much of the difficulty with the phrase “adverse environmental impact” has been with the word “adverse,” we believe the word “environmental” has too often been ignored in attempts at definition of AEI. We believe Congress intended to minimize *environmental* impact and not impact at some finer level of biological organization. We interpret population impact—as embodied in most of the definitions reviewed above—as signaling the potential for AEI.
3. The concepts of public trust resources and AEI should be separated. They have been confused in the ongoing dialogue, and this, perhaps more than anything else, has hampered consensus on a definition of AEI. Public trust resources refer to resources held in trust for the benefit of the citizens of a political entity, usually a state. Strictly interpreted, the unauthorized taking of one fish would represent a loss of public trust resources. This is a matter of societal-based policy that has no relation to AEI.

Over the last 30 years, the scientific community has attempted to define AEI on a scientific basis, i.e., based on impacts at the population level. This is consistent with the clear intent of Section 316(b) to minimize *environmental* impact. Federal 316(b) guidance should define AEI on a scientific basis. This will not preclude a state from exercising its law and policy to protect its public trust resources.
